# CHST3 Skeletal Dysplasia and GRIA3 Neurodevelopmental Disorder: A Dual Diagnosis With a Recurrent Syrian CHST3 Variant

**DOI:** 10.1155/crig/5154048

**Published:** 2026-11-08

**Authors:** Bita Hashemi, Esther Abena Frimpomaah Nkrumah

**Affiliations:** ^1^ Division of Medical Genetics Department of Pediatrics Royal University Hospital University of Saskatchewan Saskatoon Saskatchewan Canada usask.ca

**Keywords:** *CHST3*, dual diagnosis, founder variant, *GRIA3*, neurodevelopmental disorder, skeletal dysplasia, whole exome sequencing

## Abstract

*CHST3*‐related skeletal dysplasia is a rare autosomal recessive skeletal disorder characterized by disproportionate short stature, progressive skeletal abnormalities, and congenital joint dislocations, typically with preserved cognitive function. We report a Syrian boy presenting with severe skeletal dysplasia, epilepsy, profound developmental delay, and intellectual disability. Clinical evaluation and trio exome sequencing identified a homozygous recurrent pathogenic variant in *CHST3* and a de novo likely pathogenic splice‐site variant in *GRIA3*. The skeletal manifestations, including the pattern of dysplasia, were consistent with *CHST3*‐related disease. However, the severity of the neurodevelopmental phenotype and epilepsy were not explained by *CHST3* deficiency alone and were more consistent with a *GRIA3*‐related neurodevelopmental disorder. This case illustrates the importance of considering dual molecular diagnoses when a patient’s phenotype extends beyond the expected clinical spectrum of a known genetic condition. Furthermore, it provides additional evidence supporting the pathogenicity of a recurrent *CHST3* variant observed in individuals of Syrian ancestry, suggesting a possible founder effect. Recognition of blended phenotypes resulting from multiple genetic diagnoses is essential to avoid inappropriate expansion of disease phenotypes and to ensure accurate genetic counseling and clinical management.

## 1. Introduction

Spondyloepiphyseal dysplasia with congenital joint dislocations (SED‐JD) (MIM#143095), referred to in the 2023 nosology as “chondrodysplasia with congenital joint dislocations, CHST3‐related”, is a rare autosomal recessive skeletal dysplasia caused by biallelic pathogenic variants in *CHST3* [1], which encodes chondroitin 6‐O‐sulfotransferase‐1, a key enzyme in cartilage extracellular matrix development. The disorder is characterized by disproportionate short stature, congenital and progressive joint dislocations, clubfeet, spinal involvement, and early degenerative joint disease. Although the musculoskeletal phenotype is often severe, cognitive development is generally preserved.

Since its first description by Rajab et al. in 2004 [2], more than 80 individuals with *CHST3*‐related skeletal dysplasia have been reported, expanding both the allelic and phenotypic spectrum of the disorder [3–11]. Nevertheless, significant neurodevelopmental impairment, severe intellectual disability, and epilepsy remain atypical and should prompt consideration of an alternative or additional diagnosis.

*GRIA3* is an X‐linked gene encoding the GluA3 subunit of the AMPA glutamate receptor, a key mediator of excitatory neurotransmission in the central nervous system. Pathogenic variants in *GRIA3* are associated with a neurodevelopmental disorder characterized by developmental delay, intellectual disability, language impairment, behavioral difficulties, and epilepsy. Both gain‐of‐function and loss‐of‐function variants can disrupt AMPA receptor activity, contributing to a broad phenotypic spectrum and highlighting the important role of glutamatergic signaling in brain development and function [12, 13].

Here, we describe a boy of Syrian ancestry with classic *CHST3*‐related skeletal dysplasia and severe neurodevelopmental impairment. Trio exome sequencing identified a homozygous recurrent *CHST3* variant together with a de novo likely pathogenic *GRIA3* variant, establishing a dual molecular diagnosis. This case expands evidence supporting the pathogenicity of a recurrent *CHST3* allele observed in individuals of Syrian ancestry [9] and illustrates the importance of considering dual molecular diagnoses when the phenotype extends beyond the recognized manifestations of a single genetic disorder.

## 2. Materials and Methods

The patient was assessed in the Medical Genetics Clinic. Clinical information was obtained through direct examination, review of medical records, radiographic investigations, neuroimaging studies, and specialist consultations. Phenotypic findings were compared with published descriptions of *CHST3*‐related skeletal dysplasia and *GRIA3*‐related neurodevelopmental disorders.

Genomic DNA was extracted from peripheral blood samples obtained from the proband and both parents. Trio whole‐exome sequencing was performed in a clinically accredited laboratory using next‐generation sequencing on an Illumina platform. Coding exons and flanking intronic regions (±10 base pairs) of known genes were captured and sequenced. More than 98% of targeted bases achieved coverage greater than 20×, with a mean target coverage exceeding 100×.

Variants were analyzed using standard bioinformatic pipelines and interpreted according to ACMG/AMP guidelines [14]. Assessment incorporated population frequency databases, computational prediction tools, published literature, segregation analysis, and phenotypic correlation.

Written informed consent for publication of the clinical details and accompanying images was obtained from the patient’s parent. According to institutional policy, this case report did not require Research Ethics Board approval.

### 2.1. Clinical Report

A 12‐year‐old male was referred to our clinic for his short stature, skeletal malformation, history of global developmental delay, intellectual disability, absence of speech, inability to walk independently, and epilepsy. He was the first child of consanguineous parents (distant relatives) of Syrian descent. The couple had four previous miscarriages. He was born at term after an unremarkable pregnancy. The family had refugee status during this period of time, which limited access to his previous health records including growth parameters at birth. He was noted to have bilateral genu valgum and club feet deformity at birth. He developed progressive short stature and joint deformities with time.

Around 3 years of age, he experienced his first afebrile episode of generalized tonic‐clonic seizure. A brain MRI was reportedly performed; however, records were not available for review. He was started on antiepileptic medication. He had a few surgeries for correction of bilateral club feet. His seizures were well controlled until about a year ago, when he began having some breakthrough episodes. A brain MRI was arranged.

According to the parental report, developmental delays were apparent from early childhood; although details were not available. He never achieved independent ambulation and remained wheelchair dependent. He did not develop expressive language and remained nonverbal. By 12 years of age, he demonstrated significant cognitive impairment and was fully dependent for all activities of daily living, including feeding, dressing, toileting, and mobility. There was no history suggestive of developmental regression. At 12 years of age, his height was 85 cm (< 0.1%ile and well below −5 SD, affected by severe valgus deformity of his knees), weight was 19.5 kg (< 1%ile at approximately −3 to −4 SD) and head circumference was 50 cm (< 1%ile at −2 to −3 SD). Facial features were relatively nondysmorphic. He had a thin body habitus with generalized reduction in muscle mass. Musculoskeletal examination showed bilateral cubitus valgus, fixed elbow flexion contractures, and prominent radial heads. Severe valgus deformities of the knees, bilateral equinovarus foot deformities, and bilateral fifth‐finger camptodactyly were present. Passive forearm supination and pronation were preserved. No major cutaneous abnormalities were identified. Overall, the examination demonstrated profound disproportionate short stature, generalized muscle wasting, and severe skeletal deformities consistent with a generalized skeletal dysplasia (Figure 1).

**FIGURE 1:**
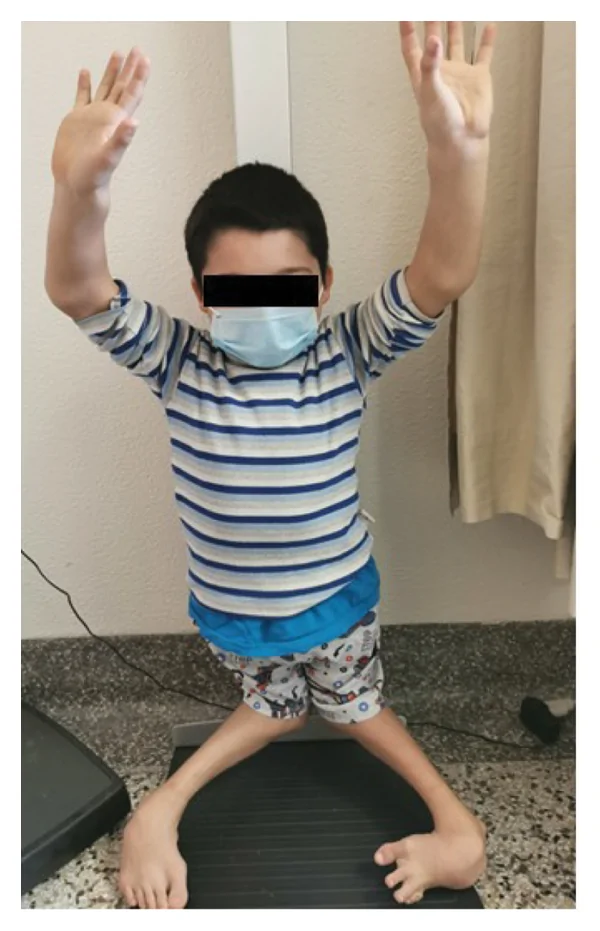
Proband at the age of 12 years old with very short stature and mesomelia, an asthenic body habitus, bilateral knee and elbow and hip dislocation, and clubfeet. Severe contractures in large joints resulting in cubitus valgus and genu valgus deformity.

### 2.2. Imaging

Skeletal radiographs demonstrated diffuse osteopenia with mesomelic limb shortening. Bilateral hip subluxation, elbow and knee malalignment, and bilateral clubfeet were present. The radiocapitellar and proximal radioulnar joints were dysplastic with anterior radial head dislocation and posteromedial ulnar displacement. Similarly, altered articulation and deformity of knees was associated with subluxation of distal femur anteriorly relative to tibia on both sides and posteriorly dislocated patella. Additional findings included platyspondyly, irregular vertebral endplates, and supernumerary proximal carpal ossification centers. Increased wormian bones were noticeable within the skull base (Figure 2).

**FIGURE 2:**
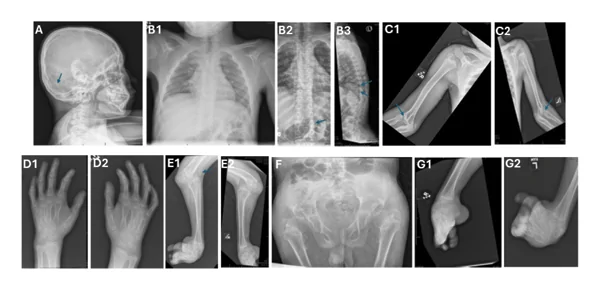
Skeletal survey showing (A) increased wormian bones (arrow) in the skull base; (B1‐B3) platyspondyly and irregular endplates of vertebral bodies (representative endplate abnormalities indicated by arrows); (C1‐C2) bilateral abnormal alignment of elbow joints (arrows); (D1‐D2) bilateral flexion contractures of fingers; (E1‐E2) fixed flexion contracture and dislocations of the bilateral knees with patella displaced posteriorly (arrow); (F) bilateral hip dysplasia; (G1‐G2) bilateral clubfeet.

Brain MRI was consistent with microcephaly and mild ventriculomegaly and bilateral hippocampal malrotation and mild white matter volume loss.

Spine MRI showed platyspondyly with multilevel vertebral endplate irregularity and cervical and lumbar spinal canal stenosis. The craniocervical junction was normal.

Echocardiography demonstrated mild aortic stenosis.

## 3. Results

Given the combination of severe skeletal dysplasia and profound neurodevelopmental impairment with epilepsy, a single unifying diagnosis was not apparent clinically, and trio exome sequencing was pursued to investigate the complex phenotype.

Trio exome sequencing identified a homozygous missense variant in *CHST3* (NM_004273.5:c.740G > C; p.Cys247Ser), inherited from both parents. The variant was initially classified by the testing laboratory as a variant of uncertain significance. However, several lines of evidence support pathogenicity, including its absence from population databases (gnomAD v.2.0) (PM2), localization within the highly conserved sulfotransferase catalytic domain (PM1), deleterious in silico predictions tools (PolyPhen‐2, SIFT, Mutation Taster, and FATHMM, PMID:2,655,599) (PP3), and the strong phenotypic concordance with *CHST3*‐related skeletal dysplasia (PP4). Furthermore, the same variant in compound heterozygous state has previously been reported in an unrelated individual with a similar skeletal phenotype [9] (PS4_supporting). Based on ACMG/AMP criteria (PM1, PM2, PP3, PP4, PS4_supporting), the variant meets the criteria for likely pathogenic classification. The CHST3 variant has been submitted to ClinVar (ClinVar ID 4688046).

In addition, a hemizygous de novo splice‐site variant in *GRIA3* (NM_007325.5: c.1293+1G > T) was identified. This variant is predicted to disrupt the canonical donor splice site and interfere with normal RNA splicing. The variant was absent from population databases and has not previously been reported. Given the established disease mechanism of loss of function in *GRIA3*‐related neurodevelopmental disorder and its de novo occurrence, applying ACMG/AMP criteria (PVS1, PS2, PM2_Supporting, and PP4), this variant was classified as likely pathogenic.

The molecular findings support a dual genetic diagnosis comprising *CHST3*‐related skeletal dysplasia and *GRIA3*‐related neurodevelopmental disorder. The *CHST3* variant explains the severe skeletal phenotype, while the *GRIA3* variant provides an explanation for his profound neurodevelopmental impairment and epilepsy.

## 4. Discussion

We describe a boy of Syrian ancestry with a severe skeletal dysplasia characterized by disproportionate short stature, congenital joint deformities, platyspondyly, and progressive skeletal involvement, together with profound neurodevelopmental impairment and epilepsy. Trio exome sequencing identified a homozygous likely pathogenic variant in *CHST3* together with a de novo likely pathogenic splice‐site variant in *GRIA3*, establishing a dual molecular diagnosis and providing a molecular explanation for his complex phenotype.

The skeletal phenotype was highly consistent with previously described *CHST3*‐related skeletal dysplasia. The close phenotypic concordance further supports the pathogenic role of the identified *CHST3* variant. Mild aortic stenosis was also present. Although cardiovascular involvement is not considered a core feature of *CHST3* deficiency, occasional reports of cardiac abnormalities suggest that cardiovascular assessment is warranted as part of the clinical evaluation of affected individuals [10, 11].

Of particular interest, the same rare *CHST3* variant has now been identified in two apparently unrelated families of Syrian ancestry, including the family reported here and the family previously described by Duz et al. [9]. While haplotype analysis was not available to investigate a shared ancestral origin, the recurrence of this variant in individuals from the same ethnic background raises the possibility of a founder allele. Identification of additional affected individuals from Syrian or neighboring populations will be important to determine the frequency and geographic distribution of this variant, and its potential founder effect.

Individuals with CHST3‐related disorder generally have normal cognitive development, and when developmental concerns are reported, they are typically mild. In contrast, our patient’s profound neurodevelopmental impairment and epilepsy appeared disproportionate to the expected CHST3‐related phenotype. The de novo likely pathogenic GRIA3 splice‐site variant provides a more plausible explanation for these neurological manifestations (Table 1).

**TABLE 1:** Comparison of the proband’s clinical manifestations with published features of CHST3‐related skeletal dysplasia and GRIA3‐related neurodevelopmental disorder to assess genotype‐phenotype correlation.

Clinical feature	CHST3‐related skeletal dysplasia [2, 3, 10]	GRIA3‐related neurodevelopmental disorder [12]	Findings in our proband
*A) Growth and skeletal findings*			
Severe disproportionate short stature	Characteristic, progressive	Not reported	Height 85 cm at 12 years below (< −5 SD)
Mesomelic/rhizomelic limb shortening	Reported	Not reported	Mesomelic shortening
Congenital joint dislocations/subluxations	Hallmark feature	Not reported	Hip, radial‐head and knee subluxation/dislocation
Clubfeet or equinovarus deformity	Common	Not reported	Bilateral clubfeet with persistent equinovarus deformities
Elbow dysplasia/contractures	Characteristic	Not reported	Dysplastic elbows, radial‐head dislocation, fixed flexion contractures
Genu valgum/progressive knee deformity	Common	Not reported	Severe bilateral genu valgum with patellar malalignment
Platyspondyly and vertebral abnormalities	Characteristic	Not reported	Platyspondyly with multilevel vertebral irregularity
Spinal canal stenosis	Reported	Not a recognized feature	Cervical and lumbar stenosis
Osteopenia	Reported	Not reported	Diffuse osteopenia

*B) Neurodevelopmental and neurologic findings*			
Global developmental delay	Usually absent or mild	Core feature	Present
Intellectual disability	Generally not expected	Core feature; severity ranges from borderline or mild to profound	Profound
Severe speech impairment	Not typical	Common	Nonverbal
Failure to achieve independent ambulation	Skeletal limitation may impair mobility	Delayed/absent walking	Never walked independently; wheelchair‐dependent
Dependence in activities of daily living	Physical dependence may occur	Common in severe cases	Fully dependent
Epilepsy	Not established	Common	Generalized tonic–clonic seizures from∼3 years
Microcephaly	Not reported	Reported in some individuals	OFC < 1^ST^ percentile
Brain MRI abnormalities	Not expected	Variable	Mild ventriculomegaly, hippocampal malrotation, white‐matter volume loss

*C) Other Features*			
Cardiac valve abnormality	occasionally reported [3, 13]	Not a defining feature	Mild aortic stenosis

*Note:* The CHST3‐related phenotype was summarized primarily from Liang et al. [2], Otaify et al. [3] and Unger et al. [10]. The GRIA3‐related phenotype was summarized from Rinaldi et al. [12], with emphasis on findings reported in individuals with loss‐of‐function variants because the canonical splice‐site variant identified in the individual described here is predicted to disrupt normal RNA splicing. Cardiac involvement in CHST3‐related skeletal dysplasia is also supported by Tuysuz et al. [13]. Suggested phenotypic attribution is based on comparison with the published phenotypes and does not imply that every feature can be assigned exclusively to one molecular diagnosis.

Abbreviations: MRI, magnetic resonance imaging; SD, standard deviation.

This case illustrates the importance of considering multilocus pathogenic variation when a patient’s phenotype is not fully explained by a single genetic diagnosis. While the skeletal manifestations are attributable to CHST3 deficiency and the neurodevelopmental features to GRIA3‐related disease, some aspects of the clinical presentation, particularly severe functional impairment and lack of independent ambulation, likely reflect the combined effects of both disorders.

Similar blended phenotypes resulting from multilocus pathogenic variation have been reported in individuals with skeletal disorders. In a cohort of 1108 patients with skeletal deformities, Liu et al. identified eight individuals with dual molecular diagnoses, demonstrating that phenotypic complexity may arise from the coexistence of two distinct Mendelian conditions rather than from atypical manifestations of a single disorder. These observations highlight the importance of considering an additional genetic diagnosis when clinical features are not fully explained by the primary skeletal dysplasia [15].

Several limitations should be acknowledged. Functional studies were not performed to confirm the impact of either variant on protein function or RNA splicing, and haplotype analysis was not available to investigate a potential founder effect. Nevertheless, the clinical and molecular findings provide strong support for the proposed dual molecular diagnoses.

In summary, this report expands the clinical and molecular literature on CHST3‐related skeletal dysplasia by identifying a recurrent variant in a second family of Syrian ancestry, raising the possibility of a shared ancestral allele. Additional cases and haplotype studies will be required to determine whether a true founder effect exists. This case also highlights the value of comprehensive genomic analysis in individuals with complex phenotypes. Recognition of a dual molecular diagnosis allowed accurate attribution of skeletal and neurodevelopmental features to two distinct genetic disorders, thereby avoiding inappropriate expansion of the known phenotypic spectrum of either condition.

## Author Contributions

Bita Hashemi conceived the study, collected and interpreted the clinical data, drafted the manuscript, and critically revised the manuscript. Co‐author, Esther Abena Frimpomaah Nkrumah contributed to manuscript writing and critically reviewed and edited the manuscript.

## Funding

No funding was obtained for this study.

## Ethics Statement

Written informed consent for publication of clinical information and clinical photographs was obtained from the patient’s parent/legal guardian. The study was conducted in accordance with institutional and journal ethical guidelines.

## Conflicts of Interest

The authors declare no conflicts of interest.

## Data Availability

The data that support the findings of this study are available upon request from the corresponding author. The data are not publicly available due to privacy or ethical restrictions.
